# Starch from the Animal Kingdom

**Published:** 1858-12

**Authors:** 


*Starch from the Animal Kingdom.*—Dr. Pavy exhibited to the Medical Society of London (Oct. 10, 1857) some of the newly discovered amylaceous material which is obtained from the healthy liver, and which forms the source of the animal sugar. In referring to the history of the saccharine secretive function of the liver, Dr. Pavy stated that, upon the announcement of the discovery by Bernard in 1848 that sugar was formed in the animal body, it was referred to a transformative action of the liver on an albuminous constituent of the portal blood. The celebrated experiment of puncturing the floor of the fourth ventricle, and establishing diabetes, rather supported this view; for it was thought that the irritation of the nervous centre stimulated the secretory action of the liver, and led to the production of a superabundance of sugar, which, accumulating in the circulatory system, was pumped off by the kidneys, and thus appeared in the urine. The next step of information removed the direct influence of the nervous system, and showed that the production of sugar could not be regarded as analogous to secretion. In September, 1855, in fact, Bernard announced, at the Académie des Sciences, that the formation of sugar continued for as many as twenty four hours after death in a liver from which the blood had been entirely removed. If, for instance, the liver of a healthy animal be removed immediately after death, and a current of water be ejected from the portal vein through its vessels, the whole of the blood is washed out, and the orgna is also quickly deprived of the saccharine material it contained. Now, if such a liver be placed on one side and examined in a few hours' time, it will be found to have become strongly impregnated with sugar, the production of this material having continued in the exsanguine tissue of the gland. Upon this fact our knowledge has rested until within the last few months. During the early part of the summer, Dr. Pavy had noticed, whilst conducting his experiments on the *destruction* of sugar (which have led him to new and unexpected results, which he will shortly communicate to the profession), that he could isolate a material from the liver which subsequently underwent transformation into sugar; and he had recorded in his laboratory book the influence of chemical agents, such as acids, alkalies, and alcohol, on this material. He had since learnt, however, on his recent visit to Paris, that Bernard had most satisfactorily made out the nature and relations of this body, which, from the analogy presented in its chemical bearings to starch, he had called an animal amylaceous or starchy material. It had also been called glycogenic material, and this was probably the best term to apply to it at present. because it implied nothing more than we knew the substance in reality to be. The specimen of this glycogenic material which Dr. Pavy exhibited, had been procured from the liver of a dog in the following manner: The dog had for some days been submitted to a strictly animal diet, so as to preclude the introduction of any starchy material into its system from the vegetable kingdom. After killing the animal, by the destruction of the medulla oblongata, the liver was removed, and a tube firmly ligatured in the portal vein, for the purpose of passing a current of water through the vessels to wash out the blood, and at the same time remove the sugar. In half an hour's time the water which had passed through the vessels was colorless and destitute of sugar, as was also the tissue of the liver itself. The organ was now cut up into small slices, placed in an evaporating dish, boiled in the liquid which exuded from it, and subsequently strained and pressed to obtain all the liquid that was procurable. The object, in fact, was to make a decoction of the liver, which holds in solution the glycogenic material, and has, thereby, communicated to it an opalescent or a milky appearance. This was then mixed with alcohol (in the proportion of one part of the decoction to about five of the spirit), and immediately a precipitate was produced, which was collected on a filter and dried, and formed the specimen then before the fellows of the Society. The substance before him was of a grayish color, which resulted from contamination with albuminous matter. It might be made perfectly white by prolonged boiling in a solution of potash, which did not at all affect its properties. It was insoluble in alcohol and strong acetic acid. Its solution in water presented the same milky appearance as the fresh decoction of liver. It gave no reaction with the sugar tests, nor was it susceptible of undergoing fermentation. It was, however, readily convertible into dextrine and sugar by the same agents which produced this change in the starch of the vegetable kingdom. Indeed, boiling with acids and contact with diastase, saliva, pancreatic juice, blood, or any animal matter in a state of change, very readily effected its transformation into glucose, when it gave all the characteristic reactions of this substance. Dr. Pavy further stated, that by means of chemical agents and the microscope the natural seat of this material could be shown to be in the imterior of the hepatic cells.—*Lancet*, Oct. 17, 1857,—from *American Journal of the Medical Sciences.*

---

*Uterus taken from a Patient who had died, of Acute Disease of the Brain, whilst Menstruating.*— Dr. W. W. GERARD exhibited this specimen, and gave the following particulars of the case:

Elizabeth Cook, æt. 25 years; English; married 7 years ago; has had two miscarriages and one still-birth; subject lately to menorrhagia, the discharge returning profusely every two weeks. She died of apoplexy, in the Pennsylvania Hospital during one of her menstrual periods.

He was indebted to Dr. Packard for the following statement relative to the part exhibited:

All the sexual organs were greatly reddened, especially the uterus, the left ovary, and the left Fallopian tube, which latter presented an enlargement near its fimbriated extremity. The inner surface of the uterus was of a very bright red color, and the orifices of its follicular glands very percepible; the mucous membrane seemed also to have a finely papillated or viltous character.

The epithelium, being scraped off and examined under the microscope was found to consist of a mixture of columnar and squamous cells. No, evidence of any new formation could be detected.

The left Fallopian tube, carefully slit open from the end to the other, contained, in the dilatation above alluded to, a quantity of dirty red, grumous liquid, which, under the microscope, was found to consist of the ordinary ciliated columnar epithelium of the tube, and of masses of corpuscles, resembling nuclei; the form of the masses was very irregular, depending entirely upon the position assumed by the corpuscles.

At several points in the surface of each ovary, there were minute dots, like orifices, each one corresponding to a Graafian vesicle. Two of these being laid open, their contents were examined under the microscope, and found to present a few granular nucleated cells of considerable size, floating in a homogeneous liquid. Patches of extremely delicate epithelium were found in the scrapings of the lining membrane of the vesicles.—*American Journal of the Medical Sciences.*

---

*Remarks on the Treatment of Gleet by Local Remedies.**—By Wm. Otterson, M. D., of Brooklyn, N. Y.

The slight discharge from the urethra so often met with after an attack of gonorrhœa, and variously known as "chronic blennorrhagia, gleet," etc., occurs under various circumstances, and its seat, cause, and treatment, are differently given by different authors. Cooper, Vidal, Acton, Hunter, Chelius, etc., give different views of the seat and pathology of the disease, yet they all concur in the general plan of treatment, viz., stimulating and astringent injections, cubebs, terebinthinates, and speak, as a last resort, of sometimes using the bougie. We find these writers placing the seat of the disease in the prostate, Cowper's glands, the lining membrane of the urethra, the lacunæ, and in the fossa navicularis. I am disposed to think that Sir A. Cooper was nearest right, and that the glans penis and the lacunae therein are the real seat of the affection. What the precise pathological condition of the glans is, I am unable to say; but it would appear that owing to its delicate structure, and the fact of its being the most frequent seat of acute attacks, that the onus of the chronic form of the disease should be centered upon this portion of the organ; and that many of the most obstinate cases of gleet (in the absence of stricture and disease of the prostate) are dependent upon a morbid condition of the glans, and not upon any disease or change of the lining membrane of the urethra itself. My reasons for thic belief are; 1st, the amount and character of the discharge; 2d, the point of irritability upon passing an instrument; 3d, the inadequacy of injections to control the disease permanently; 4th, the failure of eternal medication or hygeine to exert any special influence over it.

1st. The discharge is usually found to be nothing more than opaque mucus, which may vary in consistence according to circumstances. This in

---

* Read before the Brooklyn Medical Society.

the morning occupies the meatus, at times almost blocking it up, and is easily removed if the patient runs the finger along the under surface of the urethra, beginning about an inch from the meatus, when the secretion escapes; after that, the urethra may be squeezed from the prostatic region to the meatus, and yet no more of the discharge be procured. 2d. In passing an instrument, when about an inch from the meatus, and corresponding with the fossa navicularis, the patient complains of pain, and after the instrument passes this point, the whole tract of the urethra beyond is free from irritability. This I have found as an almost invariable result in my cases of obstinate and long standing gleet, when there was no stricture.

3d. Injections of all kinds have utterly failed to make any permanent impression upon the discharge, though there have been remissions for a few days at different times. 4th. Internal remedies have failed to exert any perceptible influence over it.

If the lining membrane of the urethra were alone involved, the result of medication would be different; for we all know, that in acute gonorrhœa, many of what are called specific medicies, do exert a modifying and decidedly beneficial influence over the diseased tract of the canal. But in this form of gleet they fail, because their action is not adapted to the disease of this particular structure. If Cowper's glands are the seat, then the application the author makes in these cases would appear to be useless, as the remedies are not applied to the portion of the canal where they are situated.

*Treatment.*—I first examine the patient to ascertain if there is stricture, narrowing of the canal, etc., and if there are any such obstructions, the usual measures are adopted to overcome them. Sometimes this alone will effect a cure, but if the discharge continues I introduce a No. 8 metallic grooved bougie, the groove being filled either with citrine ointment or calomel; the latter I prefer as being more certain in its action. The instrument need be passed only a sufficient length up the urethra to get the point beyond the glans penis, when it should be carefully revolved several times; it should be allowed to remain in for three or five minutes, and then a probe, adapted to the curve of the instrument, should be passed down the groove; if they are then withdrawn simultaneously the whole of the calomel will be left in apposition with the parts.

This may be repeated two or three times a week at the same time that the bowels are kept soluble. Upon withdrawing the instrument a little smarting may be complained of, and perhaps a few drops of blood follow, but this is not important. If this plan of treatment is followed assiduously, you can assure your patient with much confidence that a very short time only will be required to remove every trace of the most obstinate and hitherto intractable gleet.

The writer has had quite a large number of these cases under his care, and formerly was in the habit of adopting the stereotyped practice, and have them linger from month to month, and finally to pass into other hands; but since adopting the view that *benefit can only be derived by direct alterative medication to the seat of the disease*, and pursuing the plan of treatment laid down in this paper, he has not failed to cure a single case, marked benefit arising after three or four applications, even in the most unfavorable and long standing cases.

As some authors have recommended the use of mercury, with a view to produce its specific effect upon the system, I transfer one case from my note-book, illustrating its action in this affection.

Case I.—L. W. consulted me on account of a chancre, which I cauterzed with solid nit. argent., and after giving a cathartic kept him upon blue mass and opium, until its specific action became manifest, and as there was some induration, kept up a slight mercurial action for some time by hyd. potass. and ext. sarsp. comp. This treatment being continued about two weeks, the chancre healed kindly when, for the first time, the patient informed me that he had been suffering from gleet for about eight months, which, at the time he first applied, was quite severe, but he, thinking that the medication and regimen adopted for the cure of his syphilis, would also suffice for his gleet, did not mention it till after his primary disease was cured; but finding he was not free from his old discharge, he wished further advice. I proposed the bougie, but he having been present when I had introduced it on his friend, desired that some other measure should first be tried. I consented, after giving an unfavorable prognosis. The old routine of injections, etc., was gone through, and after one month's trial he concluded to submit to the bougie. Upon examination no irritable point was found after the point of the instrument passed the fossa navicularis, no stricture or narrowing of the urethra. A perfect cure was effected in about three weeks, no relapse following.

Case II.—J. D. applied to me on account of a gleet which had troubled him about five months, the sequel of a gonnorrhœa which he had treated with injections of bals. copab. Upon examination, I found some narrowing of the canal just anterior to the bulb, with irritability at this point, and at the point corresponding with the seat of the fossa navicularis. This constriction readily gave way by the use of graduated bougies, but the gleet still continued. I treated him upon tho old plan for six months, during which time all the different remedies were tried, and strict rules of regimen enjoined. In this time there were several remissions, but nothing permanent. At last I introduced the medicated bougie twice a week, at the same time, removing entirely my dietetical restrictions. At the end of six weeks, although he daily drank large quantities of beer, he was entirely cured. Some may be disposed to think that this case required tonics and nourishing diet, but these had been given as one of the changes during the six months of the old treatment.—*New York Journal of Medicine.*

